# LncRNA LINC01857 reduces metastasis and angiogenesis in breast cancer cells via regulating miR-2052/CENPQ axis

**DOI:** 10.1515/med-2022-0525

**Published:** 2022-08-10

**Authors:** Weiwei Qian, Linlin Yang, Yi Ni, Fei Yin, Lili Qin, Yang Yang

**Affiliations:** Department of Breast Surgery, Nantong Third People’s Hospital, Nantong University, Nantong, Jiangsu Province, China; Department of Oncology, Sheyang People’s Hospital, Yancheng City, Jiangsu Province 224300, China; Department of Endoscopic Center, Affiliated Hospital of Nantong University, Nantong City, Jiangsu Province 226001, China; Department of Trauma Center, Affiliated Hospital of Nantong University, No. 20 Xisi Road, Chongchuan District, Nantong City, Jiangsu Province 226001, China

**Keywords:** breast cancer, LncRNA LINC01857, MiR-2052, CENPQ, angiogenesis, metastasis

## Abstract

Long non-coding RNAs have been confirmed closely related to the metastasis and angiogenesis of breast cancer (BC). LINC01857 can promote the growth and metastasis of BC cells. The present work focused on exploring the role of LINC01857 in BC metastasis and angiogenesis and investigating the possible mechanisms. The results showed that LINC01857 and CENPQ were highly expressed in BC tissues and cells, while miR-2052 was contrarily expressed. *In vitro* study showed that low expression of linc01857 could inhibit the migration ability and vascularization of BC cells, and mir-2052 inhibitor partially restored the effect of si-LINC01857 on the migration ability and vascularization of BC cells. Likewise, inhibition of CENPQ can partially rescue the effects of miR-2052 inhibitor on the migration ability and vascularization of BC cells. *In vivo* studies showed that down-regulation of LINC01857 notably suppressed tumor growth and angiogenesis in nude mice. The miR-2052 inhibitor partially restored the effects of si-LINC01857. CENPQ suppression partially rescued the effects of the miR-2052 inhibitor. To conclude, LINC01857/miR-2052/CENPQ is the potential novel target for BC treatment.

## 1 Introduction

Breast cancer (BC) is the most common female malignancy worldwide, with the second leading cause of cancer-related death [1,2]. According to the presence or absence of estrogen or progesterone receptors and molecular markers of human epidermal growth factor 2 (ERBB2), BC is classified into three main subtypes: ERBB2 negative (ERBB2−), ERBB2 positive (ERBB2+), and triple-negative (Triple-Neg) [3]. In recent years, the BC incidence rate has increased yearly, which is very harmful to women’s physical and mental health [4]. At present, the treatment methods of BC mainly include surgery, chemotherapy, molecular therapy, and so on [5,6]. Although the clinical research on BC treatment has made rapid progress, the postoperative recurrence rate of BC is still very high, and the prognosis of BC patients has not achieved the expected effect with less than 10% 5-year survival rate [7]. Moreover, the postoperative recurrence of BC is closely associated with angiogenesis of BC, and high-density tumor neovascularization suggests a poor prognosis [8]. Therefore, study on the regulatory mechanisms of BC angiogenesis plays an essential role in preventing and treating BC.

Tumor angiogenesis refers to the generation of new blood vessels from the existing blood vessels, which is one of the crucial characteristics of tumors [9]. Tumor cells, tumor environmental stromal cells, extracellular matrix, vascular endothelial cells, and the cellular transmitters synthesized and released by them participate in this process [10]. When tumors reach a particular size, tumor cells and stromal cells in the tumor microenvironment create stimulating factors that cause endothelial cell proliferation and migration, resulting in the formation of a new vessel for nourishment and blood supply [11]. Tumor angiogenesis not only promote tumor growth but also help tumor cells fall off, enter blood vessels, or spread to the adjacent matrix, creating conditions for tumor invasion and metastasis [12]. Researchers have found that the higher the microvessel density in tumor tissues, the greater the metastatic potential and the worse the prognosis [13]. Over the years, many outstanding achievements have been made in researching the mechanisms of tumor angiogenesis [14]. However, at present, the relevant regulatory agencies of BC angiogenesis process are not completely clear.

Long non-coding RNAs (lncRNAs) are non-coding RNAs with a length of more than 200 nt [15]. They can participate in genome regulation at pre-transcriptional level, transcription level, and post-transcriptional level through epigenetic silencing and splicing regulation. Interaction between lncRNAs and microRNAs (miRNAs), lncRNAs and mRNAs, or lncRNAs and proteins, widely participates in the regulation of individual growth and development, cell apoptosis, proliferation, and differentiation [16–18]. The imbalance of lncRNA levels is related to many cancers, including BC. For example, LINC02273 was up-regulated both within BC tissues and cells. LINC02273 knockdown inhibited BC metastasis *in vitro* and *in vivo* accomplished by modulating AGR2 [19]. HOTAIR down-regulation repressed propagation and metastasis and induced apoptosis through regulating the miR-20a-5p/HMGA2 axis [20]. MEG3 was decreased within BC tissues and cells. Up-regulation of MEG3 significantly inhibited angiogenesis *in vitro* and *in vivo* by negatively regulating AKT signaling [21]. LINC00968 was reduced in BC tissues. LINC00968 up-regulation inhibited BC cell proliferation, metastasis, and tube formation abilities by inhibiting miR-423-5p [22].

LINC01857, as a pro-cancer factor, has been confirmed related to the development of many cancers. For example, LINC01857 levels were increased within diffuse large B-cell lymphoma (DLBCL) tissues and cells. LINC01857 down-regulation inhibited the proliferation and cell cycle arrest but promoted the apoptosis of DLBCL cells [23]. LINC01857 was increased within glioma and negatively correlated with the survival rate in glioma patients. LINC01857 down-regulation repressed glioma proliferation and invasiveness *in vitro* and *in vivo* [24]. Moreover, LINC01857 levels were up-regulated within BC tissues and cells. LINC01857 knockdown inhibited the growth and metastasis of BC cells [25]. However, the detailed role and possible mechanisms of LINC01857 on BC metastasis and angiogenesis remain unclear.

The present work focused on elucidating the levels of LINC01857 in BC tissues and cells and exploring the possible mechanisms of LINC01857 in functional activities of BC cell metastasis and angiogenesis, which will imply the potential of LINC01857 to be a novel potential treatment target in treating BC.

## 2 Materials and methods

### 2.1 Collection of BC tissues

Thirty BC tissues (including nine cases of ERBB2+, six cases of ERBB2−, and 15 cases of Triple-Neg) and 30 corresponding tissues were collected from the Affiliated Hospital of Nantong University. Each participant provided written informed consent for participation.

### 2.2 Cell lines

MCF10A cells, BC cells (MCF-7, BT474, SKBr-3, ZR-75-30, and MDA-MB-231), and HUVEC were provided by ATCC (USA) and cultured within dulbecco's modified eagle medium (DMEM) (Thermo Fisher Scientific, USA) containing 10% FBS (Thermo Fisher Scientific, USA) at 37°C and 5% CO_2_ conditions.

### 2.3 Cell transfection

si-LINC01857 (5′-CAGUGUUCAUGAAAGCAAA-3′), si-CENPQ (5′-GGUAGAGACCACAGAGU UA-3′), negative empty vectors (si-NC) (5′-GCTTCGCGCCGTAGTCTTA-3′), NC inhibitor (5′-CAG UACUUUUGUGUAGUACAA-3′)/miR-2052 inhibitor (5′-AGUUCAAAGUUACCAGCUA-3′), and NC mimic (5′-UUUGUAC UACACAAAAGUACUG-3′)/miR-2052 mimic (5′-UGUUUUGAUAAC AGUAAUGU-3′) were obtained from Ambion (Austin, USA) and transfected into BC cells (5 × 10^5^) maintained within the six-well plates using Lipofectamine 3000 (Invitrogen, USA). Transfection efficiency was examined by qRT-PCR after 48 h.

### 2.4 Angiogenesis experiment

Transfected MDA-MB-231 cells (3 × 10^5^/well) were placed in 24-well plates, and the tumor-conditioned medium (TCM) was collected after 24 h. Next, HUVEC was inoculated into 24-well plates pre-coated with Matrigel and incubated with the above-collected medium. Then cells were fixed with 4% paraformaldehyde and imaged via a light microscope (Nikon, Japan).

### 2.5 Wound healing assay

Transfected cells (5 × 10^5^/well) were placed into six-well plates at the appropriate density. After cells reached 80% confluence, a wound was scratched. Cell images at 0 and 48 h were photoed with a light microscope (Nikon, Japan) (200×).

### 2.6 Transwell analysis

For invasion assays, Matrigel was solved with serum-free DMEM, and uniformly covered on the Transwell chamber for 1 h at 37°C. For migration and invasion assays, BC cells from the different groups were seeded into upper chambers at appropriate density. Medium containing 10% FBS was added into lower chambers. Following a 48-h incubation period at 37°C at room temperature, migrated and invaded cells were stained with crystal violet (0.1%) and photoed with a light microscope (Nikon, Japan) (200×).

### 2.7 qRT-PCR analysis

TRIzol reagents (Beyotime Biotechnology, Shanghai, China) were utilized to extract total RNA from gastric cancer (GC) tissues and cells. Later, cDNA was prepared from total RNA by TaqMan one-step reverse transcription (Applied Biosystems, USA) through reverse transcription. The ABI Prism 7500 system (Applied Biosystems, USA) was used for qRT-PCR following specific protocols. We measured the relative levels of LINC01857, miR-2052, CENPQ, U6, and β-actin by 2^−ΔΔCt^ method. U6 and β-actin served as the endogenous references. Primers used in this work are shown in Table 1.

**Table 1:** Primer sequences

Gene name	Primer sequences
LINC01857	F:5′-CAGGACTCCATTAAGGACTC-3′
	R:5′-AACATCGATGTGTCCCAGGA-3′
miR-2052	F:5′-TCGGCAGGUGUUUUGAUAAC-3′
	R:5′-CAGTGCGTGTCGTGGAGT-3′
CENPQ	F:5′-AAGGGCACGAGACAAAGCTAA-3′
	R:5′-ACCTCACTTGCCAGAATCTGA-3′
U6	F: 5′-CTCGCTTCGGCAGCACA-3′
	R:5′-AACGCTTCACGAATTTGCGT-3′
β-actin	F:5′-CATGTACGTTGCTATCCAGGC-3′
	R:5′-CTCCTTAATGTCACGCACGAT-3′

### 2.8 Western blot

Protein was isolated from BC cells and measured through the BCA kit (Beyotime Biotechnology, China). Protein was extracted using 12% SDS-PAGE and then shifted into poly(vinylidene fluoride) membranes (Millipore, USA), which were incubated using 5% skimmed milk, followed by overnight incubation with primary antibodies under 4°C. After rinsing the membranes, they were incubated for 1 h using HRP-labeled secondary antibody (1:4,000, SA00004-10, Proteintech, China) under ambient temperature. Finally, the enhanced chemiluminescence kit (ECL, Millipore, Bedford, USA) was utilized to observe protein blots, whereas ImageJ software (NIH, version 4.3) was adopted for quantification. All primary antibodies used in this study included anti-VEGF (1:2,000, 19003-1-AP, Proteintech, China), anti-CD31 (1:2,000, 11265-1-AP, Proteintech, China), anti-MMP-2 (1:2,000, 10373-2-AP, Proteintech, China), anti-MMP-9 (1:2,000, 10375-2-AP, Proteintech, China), anti-CENPQ (1:2,000, ab105742, Abcam, USA), and anti-β-actin (1:2,000, 66009-1-Ig, Proteintech, China) with β-actin being the endogenous control.

### 2.9 Dual-luciferase reporter assay

This study sub-cloned LINC01857 or CENPQ1 WT/MUT to the pmirGLO dual-luciferase vectors (Promega, USA) for generating pmirGLO-LINC01857 or CENPQ WT/MUT to co-transfect into cells with NC mimic or miR-2052 mimic. After co-transfected for 48 h, the relative luciferase activity was measured (Promega, USA).

### 2.10 Xenograft model

Balb/c nude mice (6 weeks old, 18–22 g) were provided from Animal welfare and experimental procedures and were complied with national guidelines. Briefly, 100 µL of 2 × 10^6^ MDA-MB-231 cells from different groups were injected into the axilla and routinely monitored and sacrificed on Day 28. At the end of time, the tumors were removed, and the length and width of the tumors were manually monitored using a Vernier caliper.

### 2.11 H&E staining assay

Tumor tissues were fixed for 24 h with 4% paraformaldehyde and then made into 4 μm slices. Afterward, the slices were differentiated for 50 s with 1% hydrochloric acid ethanol and re-stained for 2 min with 1% eosin. Finally, the pieces were photographed to observe pathological changes using a light microscope (Nikon, Japan) (200×).

### 2.12 Immunohistochemistry assay

The slices from different groups were sealed for 30 min with 3% bovine serum protein at 37°C. Then slides were incubated overnight with anti-VEGF (1:500, 19003-1-AP, Proteintech, China) and anti-CD31 (1:500, 11265-1-AP, Proteintech, China). Afterward, slices were incubated with a second antibody for 60 min at room temperature and then incubated for 15 min at room temperature with 4′,6-diamidino-2-phenylindole. Fields were photographed in each slice using a Leica TCS SP5 microscope (Leica Microsystems, Wetzlar, Germany).

### 2.13 Statistical analysis

Data were analyzed using GraphPad Prism 5.0 and presented in the form of mean ± SD. Differences between groups were compared by one-way ANOVA and Turkey’s poc host. *P* < 0.05 stood for statistical significance.


**Ethics approval and consent to participate:** The experimental protocol was established, according to the ethical guidelines of the Helsinki Declaration and was approved by the Ethics Committee of Affiliated Hospital of Nantong University. Each participant provided written informed consent for participation.

## 3 Results

### 3.1 LINC01857 levels are up-regulated in BC tissues and cells

**Figure 1:**
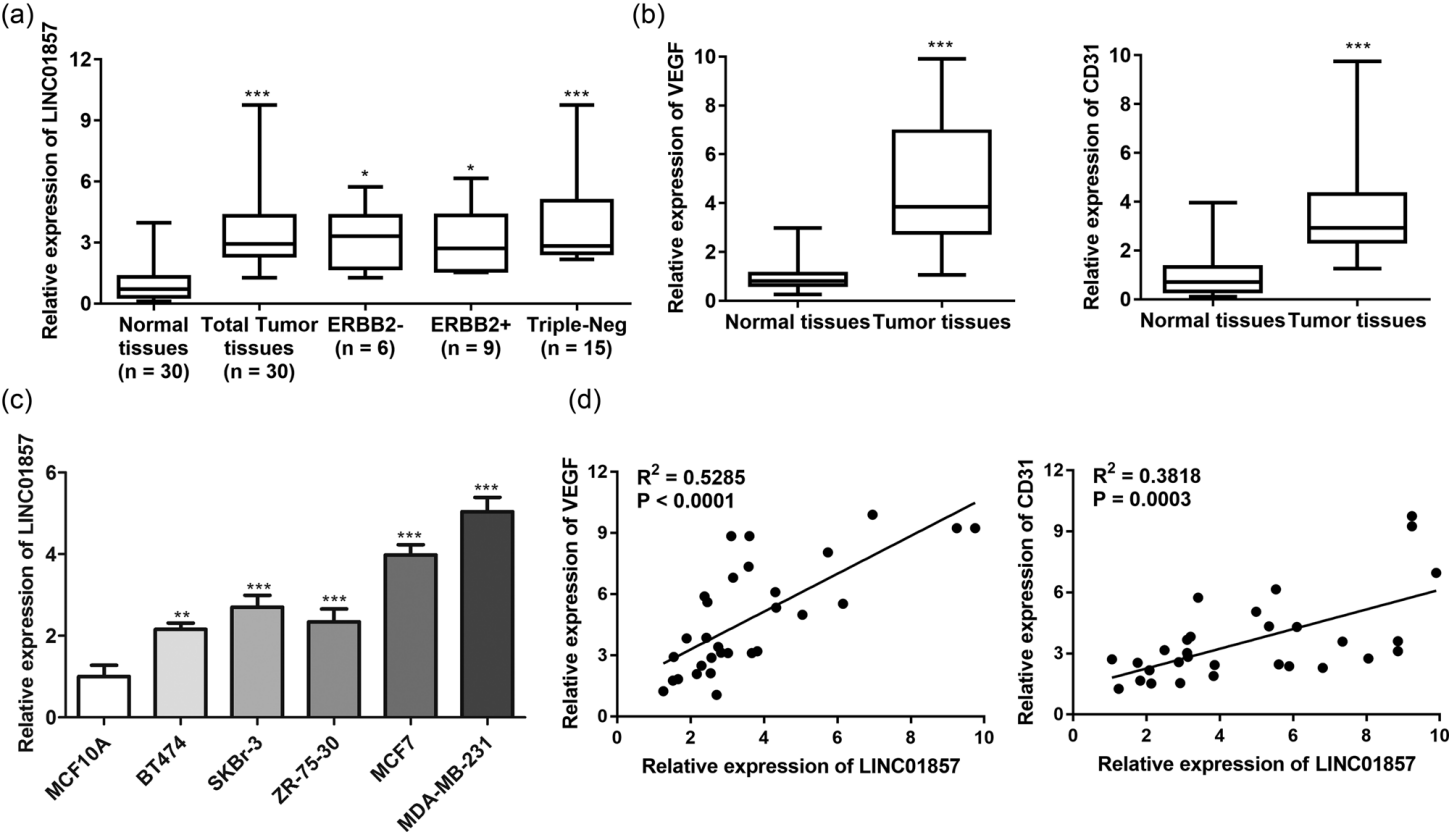
LINC01857 levels are increased in BC tissues and cells: (a) expression of LINC01857 in total tumor tissues, and the three isoforms (ERBB2−, ERBB2+, and Triple-Neg) were determined by qRT-PCR; (b) VEGF and CD31 levels in BC tissues and (c) cells were detected by qRT-PCR assay; (d) the correlation of VEGF or CD31 with LINC01857 was analyzed by correlation analysis. ^**^ *P* < 0.01 and ^***^ *P* < 0.001 vs normal tissues or MCF10A cells.

LINC01857 levels within BC patient tissue were detected via qRT-PCR analysis. LINC01857 was highly expressed in BC tissues and tissues of the three BC subtypes (ERBB2−, ERBB2+, and Triple-Neg) were compared with normal tissues (Figure 1a), and VEGF and CD31 (Figure 1b) highly expressed in BC tissues were compared with normal tissues. LINC01857 expressions *in vitro* were also investigated through qRT-PCR analysis. According to Figure 1c, LINC01857 levels were up-regulated in breast cells, especially in MDA-MB-231 cells. Additionally, Figure 1d indicates that VEGF and CD31 were both significantly positively correlated with LINC01857 expression.

### 3.2 LINC01857 down-regulation represses metastasis of BC cells and angiogenesis of HUVEC

**Figure 2:**
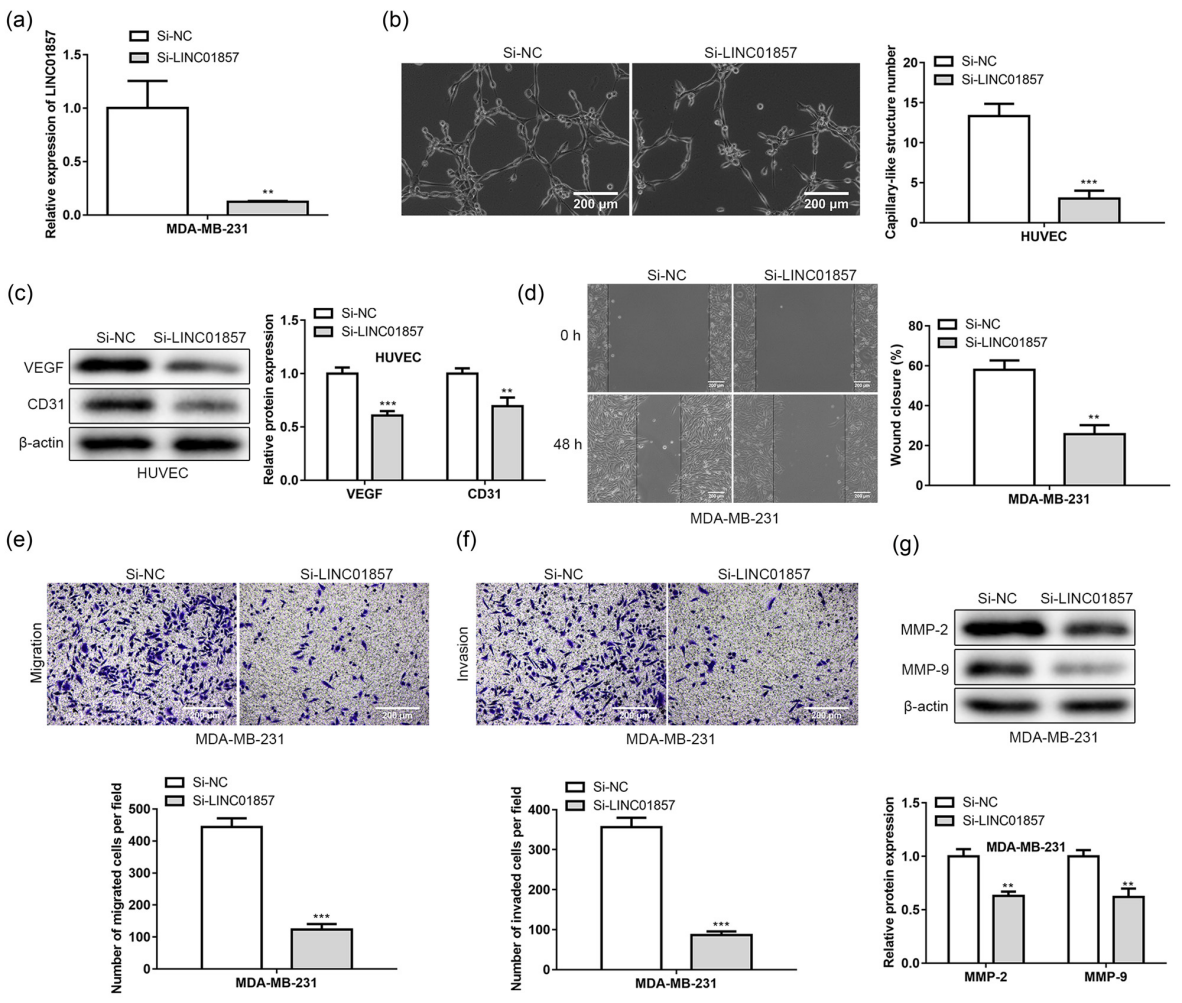
LINC01857 down-regulation inhibits metastasis of BC cells and angiogenesis of HUVEC: (a) qRT-PCR analysis was performed to evaluate the LINC01857 expressions in MDA-MB-231 transfected with si-LINC01857; (b) angiogenesis experiment was used to evaluate the effects of si-LINC01857 on the tube formation of HUVEC cells; (c) levels of angiogenesis-related proteins, including VEGF and CD31 in TCM-treated HUVEC cells, were evaluated by western blotting assay. The effects of si-LINC01857 on MDA-MB-231 cells metastasis were detected by (d) wound healing and Transwell, (e) migration, and (f) invasion analysis. (g) The levels of metastasis-related proteins, including MMP-2 and MMP-9 in MDA-MB-231 cells transfected with si-LINC0185, were evaluated by western blotting assay. ^**^ *P* < 0.01 and ^***^ *P* < 0.001 vs si-NC group.

Si-NC and si-LINC01857 were transfected into MDA-MB-231. Transfection efficiency was evaluated by qRT-PCR as displayed in Figure 2a. It showed that LINC01857 expression was obviously decreased in MDA-MB-231 cells transfected with si-LINC01857. Then angiogenesis experiment was conducted for assessing the effects of si-LINC01857 on angiogenesis of HUVEC cells. According to Figure 2b, LINC01857 down-regulation inhibited the ability of angiogenesis of HUVEC cells, and the capillary-like structure number was significantly reduced. In addition, a western blot was conducted for determining angiogenesis-related protein levels affected by si-LINC01857. Figure 2c reveals that LINC01857 low-expression suppressed the VEGF and CD31 expressions in HUVEC cells.

Moreover, we carried out wound healing and Transwell analysis to assess si-LINC01857 on metastasis of MDA-MB-231 cells. The results of the wound healing assay showed that low expression of LINC01857 could reduce the rate of wound healing of MDA-MB-231 cells (Figure 2d). The results of Transwell assay showed that the number of migratory (Figure 2e) and invasive cells was significantly decreased in MDA-MB-231 cells with low expression of LINC01857 (Figure 2f). Furthermore, a western blot was carried out for determining the levels of metastasis-related proteins affected by si-LINC01857. Figure 2g displays that LINC01857 down-regulation decreased MMP-2 and MMP-9 expressions in MDA-MB-231 cells. These data indicated that LINC01857 down-regulation inhibited metastasis of BC cells and angiogenesis of HUVEC.

### 3.3 Regulatory relationship between LINC01857 and miR-2052 in BC

**Figure 3:**
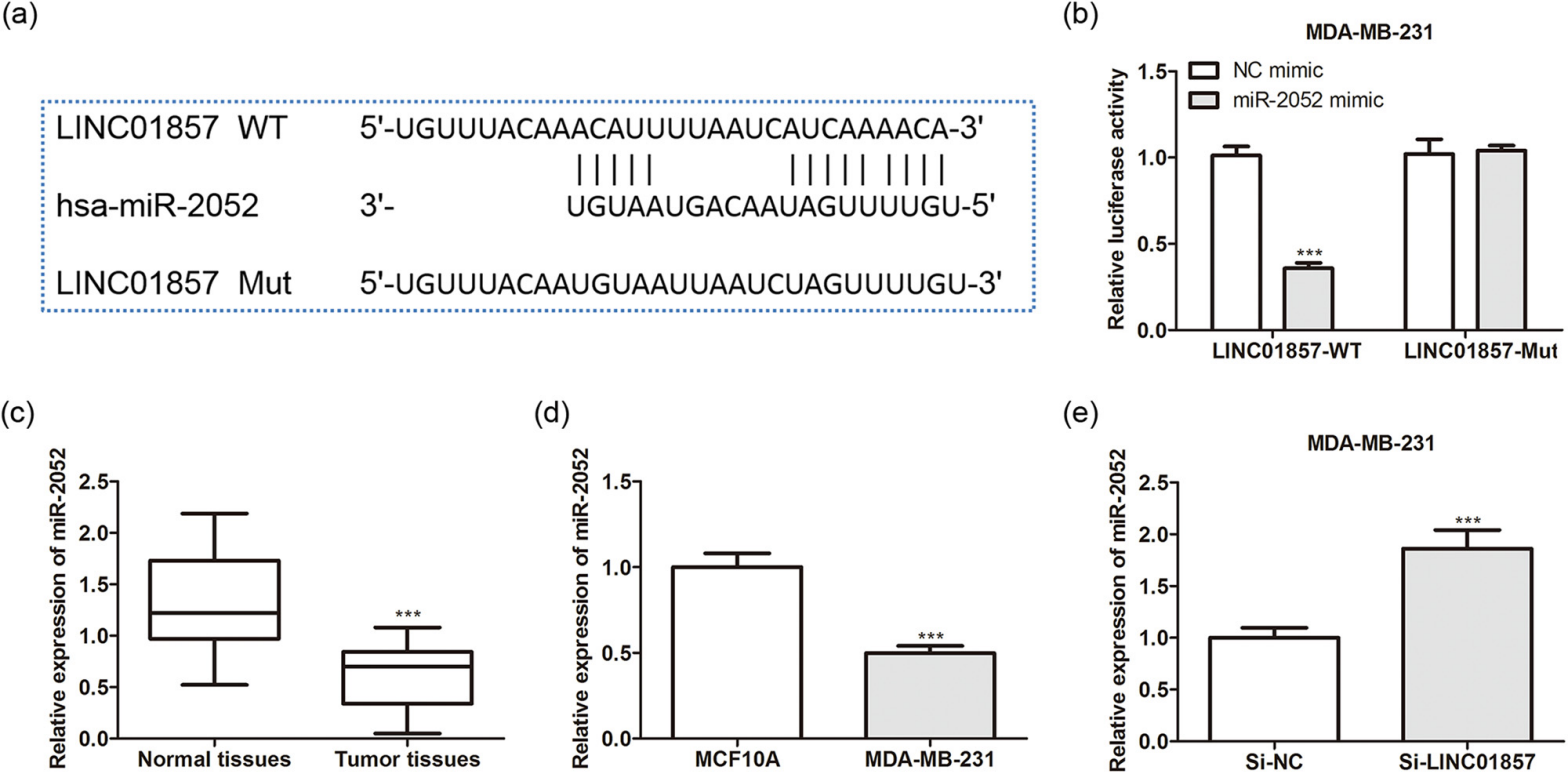
Regulatory relationship between LINC01857 and miR-2052 in BC: (a) the binding sites between LINC0185 and miR-2052; (b) the interaction between LINC0185 and miR-2052 was evaluated by a dual-luciferase reporter assay. ^***^ *P* < 0.001 vs NC mimic group. MiR-2052 levels in BC (c) tissues and (d) cells were detected by the qRT-PCR assay. ^***^ *P* < 0.001 vs normal tissues or MCF10A cells. (e) MiR-2052 levels in BC cells transfected with si-LINC01857 were determined by qRT-PCR assay. ^***^ *P* < 0.001 vs si-NC group.

In order to ascertain the possible regulatory mechanisms of LINC01857, Bioinformatics (https://www.bioinformatics.com.cn) and RNAcentral (https://rnacentral.org/), two bioinformatics tools were utilized, and dual-luciferase reporter analysis further validated the association of LINC01857 and miR-2052 (Figure 3a and b). Additionally, miR-2052 levels were reduced within BC tissues and cells via qRT-PCR analysis (Figure 3c and d). Moreover, miR-2052 levels were up-regulated in BC cells transfected with si-LINC01857 (Figure 3e). These data indicated that LINC01857 negatively regulated miR-2052 in BC.

### 3.4 MiR-2052 targets CENPQ in BC

**Figure 4:**
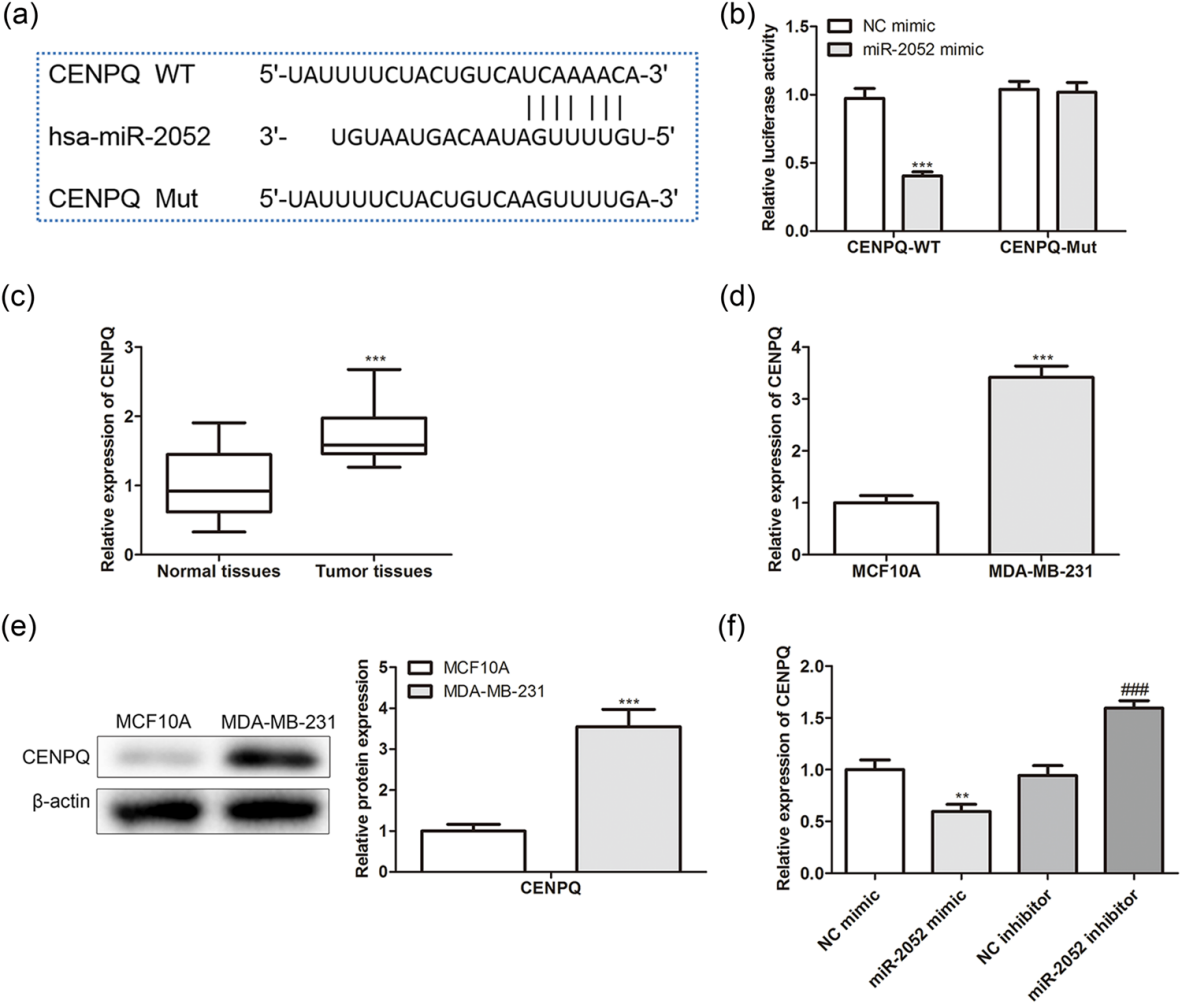
MiR-2052 targets CENPQ in BC: (a) the binding sites between miR-2052 and CENPQ; (b) the interaction between miR-2052 and CENPQ was evaluated by a dual-luciferase reporter assay. ^***^ *P* < 0.001 vs NC mimic group. (c) CENPQ levels in BC tissues were detected by the qRT-PCR assay. ^***^ *P* < 0.001 vs normal tissues. CENPQ levels in BC cells were detected by (d) qRT-PCR and (e) western blot assays. ^***^ *P* < 0.001 vs MCF10A cells. (f) CENPQ levels in BC cells transfected with miR-2052 mimic/inhibitor were determined by qRT-PCR assay. ^**^ *P* < 0.01 vs NC mimic group and ^###^ *P* < 0.001 vs or NC inhibitor group.

Moreover, Targetscan (https://www.targetscan.org/vert_72/) bioinformatics tools were used to explore the possible miR-2052 targets, and dual-luciferase reporter analysis verified the target relationship between miR-2052 and CENPQ (Figure 4a and b). Additionally, CENPQ levels were increased within BC tissues and cells via qRT-PCR and western blot (Figure 4c–e). Moreover, CENPQ levels were decreased in miR-2052 mimic-transfected BC cells and were increased in miR-2052 inhibitor-transfected BC cells via qRT-PCR (Figure 4f). These data indicated that CENPQ was the direct target of miR-2502 in BC.

### 3.5 MiR-2052/CENPQ mediates LINC01857 functions on angiogenesis and metastasis of BC cells

**Figure 5:**
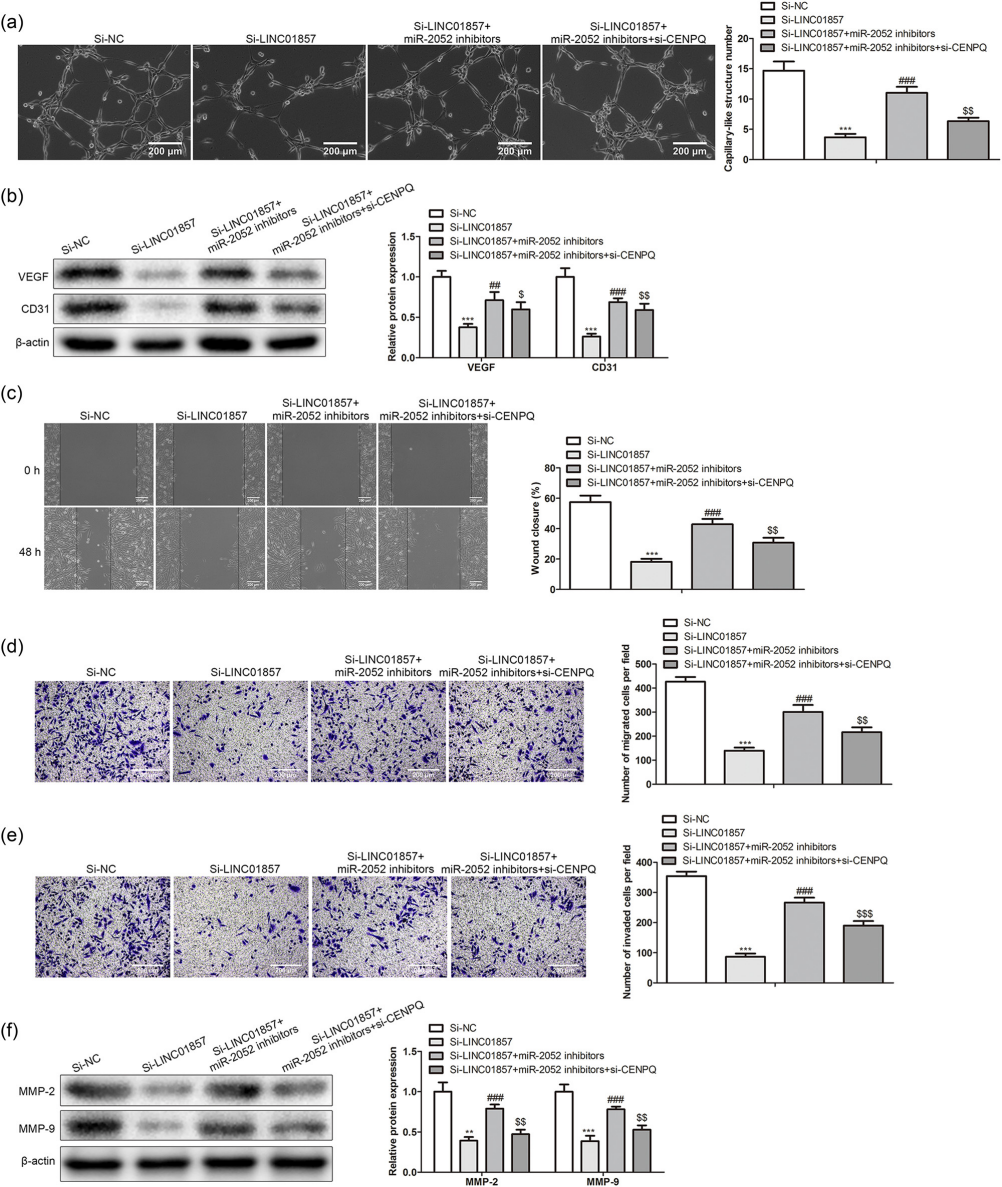
MiR-2052/CENPQ mediates LINC01857 functions on angiogenesis and metastasis of BC cells: (a) angiogenesis experiment was used to evaluate the angiogenesis of BC cells after transfection; (b) western blot was used to evaluate the angiogenesis-related proteins. The metastasis of BC cells after transfection was determined by (c) wound healing and Transwell, (d) migration, and (e) invasion analysis. (f) Western blot was used to evaluate the metastasis-related proteins. ^**^ *P* < 0.01, ^***^ *P* < 0.001 vs si-NC group, ^##^ *P* < 0.01, ^###^ *P* < 0.001 vs si-LINC01857 group, ^$^ *P* < 0.05, ^$$^ *P* < 0.01, and ^$$$^ *P* < 0.001 vs si-LINC01857 + miR-2052 inhibitor group.

To further investigate whether LINC01857 exhibited its functional effects on BC cells by regulating the miR-2052/CENPQ axis, a series of rescue assays were performed. According to the angiogenesis experiment, miR-2052 inhibitor partially restored the effects of si-LINC01857, while si-CENPQ partially rescued the effects of the miR-2052 inhibitor on the angiogenesis abilities of BC cells (Figure 5a). Similarly, miR-2052 inhibitor increased the levels of VEGF and CD31 induced with si-LINC01857, and CENPQ down-regulation exhibited the opposite effects (Figure 5b). Based on wound healing and Transwell analysis, down-regulation of miR-2052 showed the opposite role of si-LINC01857 on BC migration and invasion, while si-CENPQ partially rescued the effects of miR-2052 inhibitor (Figure 5c–e). As expected, MMP-2 and MMP-9 protein levels were up-regulated in the miR-2052 inhibitor-transfected group relative to the si-LINC01857 group. At the same time, they were down-regulated in a si-CENPQ group compared with a miR-2052 group (Figure 5f). These data indicated that miR-2052/CENPQ mediated LINC01857 functions on angiogenesis and metastasis of BC cells.

### 3.6 LINC01857 down-regulation inhibits BC tumor growth *in vivo*

**Figure 6:**
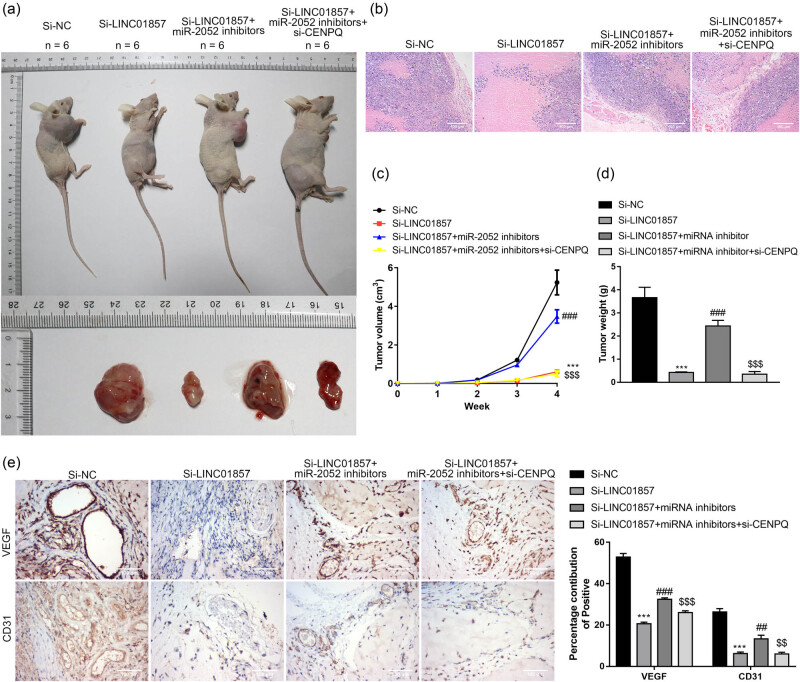
LINC01857 down-regulation inhibits BC tumor growth *in vivo*: (a) the representative pictures of mice and tumor tissues are shown (*n* = 6); (b) histological changes of tumors from each group were determined by HE staining; (c) the volumes and (d) weight of tumors from each group were determined; (e) the protein expressions of VEGF and CD31 in tumors from each group were detected by immunohistochemical analysis. ^***^ *P* < 0.001 vs si-NC group, ^###^ *P* < 0.001 vs si-LINC01857 group, and ^$$$^ *P* < 0.001 vs si-LINC01857 + miR-2052 inhibitor group.

To further verify the influence of LINC01857 in BC tumor growth *in vivo*, transfected MDA-MB-231 cells were injected into nude mice. According to Figure 6a–c, LINC01857 down-regulation inhibited the tumor volume and weight (Figure 6d). Moreover, HE staining data suggested that the tumor cells in the si-LINC01857 group were lightly stained with less mitosis, and more tumor cells were swollen, degenerated, and necrotic (Figure 6b). Furthermore, compared to the si-NC group, LINC01857 down-regulation inhibited the VEGF and CD31 expressions (Figure 6e). Interestingly, miR-2052 inhibitor partially restored the effects of si-LINC01857, and si-CENPQ exhibited the opposite effects compared with miR-2052 inhibitor. These data indicated that LINC01857 down-regulation inhibited BC tumor growth *in vivo* by regulating the miR-2052/CENPQ axis.

## 4 Discussion

Many lncRNAs exerted a crucial role in metastasis and angiogenesis of BC. RAB11B-AS1 over-expression enhanced angiogenic factor expressions (VEGFA and ANGPTL4) [26]. Besides, PCDHB17P knockdown suppressed metastasis [27]. AC073352.1 down-regulation inhibited the metastasis and angiogenesis of BC cells [28]. These findings indicate that lncRNAs exhibit an essential impact on BC metastasis and angiogenesis. In our study, LINC01857 levels were increased within BC tissues and cells. LINC01857 down-regulation repressed the migration, invasion, and angiogenesis of BC cells. Moreover, LINC01857 down-regulation inhibited the growth of BC *in vivo* and suppressed the VEGF and CD31 expressions. These data were consistent with previous studies [25], suggesting that LINC01857 might act as an oncogene in BC metastasis and angiogenesis.

LncRNAs have been commonly acknowledged serving as ceRNAs through regulating miRNA expressions in cancer metastasis and angiogenesis. LINC01410 down-regulation inhibited GC metastasis and angiogenesis by negatively regulating miR-532-5p [29]. H19 silence suppressed the proliferation, migration, and angiogenesis of glioma cells by modulating miR-138 [30]. Moreover, knockdown of DANCR impaired ovarian cancer tumor growth through inhibition of tumor angiogenesis via up-regulating miR-145 [31]. In our study, we conducted online bioinformatics to retrieve miRNAs interacting with LINC01857, and miR-2052 was selected, which was further confirmed by dual-luciferase reporter analysis. It has been reported that miR-2052 levels were reduced in hepatocellular carcinoma (HCC) tissues and cells. MiR-2052 over-expression suppressed the proliferation and metastasis of HCC cells *in vitro* and suppressed the xenograft tumor growth *in vivo* [32]. Moreover, miR-2052 levels were down-regulated in oral cancer tissues [33]. Our data showed that miR-2052 levels were decreased within BC tissues and cells and negatively associated with LINC01857. Functionally, miR-2052 inhibitor partially restored the impacts of si-LINC01857 on metastasis and angiogenesis of BC both *in vitro* and *in vivo.* Based on database analysis, LINC01857 expression was positively associated with CENPQ. 3′-UTR region of CENPQ contained complementary sites to miR-2052, which suggested the potential of CENPQ as the miR-2052 target, as validated through dual-luciferase reporter analysis. Recent studies indicated that CENPQ levels were increased in HCC, and closely associated with cell division cycle-associated gene alterations [34]. Thus, rescue experiments were performed to explore the role of CENPQ on miR-2052 inhibitor on metastasis and angiogenesis of BC mediated with si-LINC01857. As expected, CENPQ suppression partially restored miR-2052 inhibitor functions in metastasis and angiogenesis of BC mediated with si-LINC01857 both *in vivo* and *in vitro*.

Taken together, LINC01857 and CENPQ levels were increased, while miR-2052 levels were decreased in BC tissues and cells. Moreover, *in vitro*, si-LINC01857 inhibited both metastasis and angiogenesis of BC cells, and *in vivo*, we found that si-linc01857 inhibited tumor growth and vascularization, possibly through regulation of miR-2052/CENPQ, providing an experimental basis that LINC01857 potentially served as the novel marker for BC treatment.

However, this experiment still has some limitations, for example, the cells studied were too single and the corresponding study of cell migration was not performed at the *in vivo* level. To better deeply investigate the role of LINC01857 in BC, studies from more levels are still needed.
